# Development and Validation of a Nine-Redox-Related Long Noncoding RNA Signature in Renal Clear Cell Carcinoma

**DOI:** 10.1155/2020/6634247

**Published:** 2020-12-28

**Authors:** Xia Qi-Dong, Xun Yang, Jun-Lin Lu, Chen-Qian Liu, Jian-Xuan Sun, Cong Li, Shao-Gang Wang

**Affiliations:** Department and Institute of Urology, Tongji Hospital, Tongji Medical College, Huazhong University of Science and Technology, No. 1095 Jiefang Avenue, 430030 Wuhan, China

## Abstract

**Background:**

Redox plays an essential role in the pathogeneses and progression of tumors, which could be regulated by long noncoding RNA (lncRNA). We aimed to develop and verify a novel redox-related lncRNA-based prognostic signature for clear cell renal cell carcinoma (ccRCC).

**Materials and Methods:**

A total of 530 ccRCC patients from The Cancer Genome Atlas (TCGA) were included in this study. All the samples were randomly split into training and test group at a 1 : 1 ratio. Then, we screened differentially expressed redox-related lncRNAs and constructed a novel prognostic signature from the training group using the least absolute shrinkage and selection operation (LASSO) and COX regression. Next, to verify the accuracy of the signature, we conducted risk and survival analysis, as well as the construction of ROC curve, nomogram, and calibration curves in the training group, test group, and all samples. Finally, the redox gene-redox-related lncRNA interaction network was constructed, and gene set enrichment analysis (GSEA) was performed to investigate the status of redox-related functions between high/low-risk groups.

**Results:**

A nine-redox-related lncRNA signature consisted of *AC025580.3*, *COLCA1*, *AC027601.2*, *DLEU2*, *AC004918.3*, *AP006621.2*, *AL031670.1*, *SPINT1-AS1*, and *LAMA5-AS1* was significantly associated with overall survival in ccRCC patients. The signature proved efficient, and thus, a nomogram was successfully assembled. In addition, the GSEA results demonstrated that two major redox-related functions were enhanced in the high-risk group ccRCC patients.

**Conclusions:**

Our findings robustly demonstrate that the nine-redox-related lncRNA signature could serve as an efficient prognostic indicator for ccRCC.

## 1. Introduction

Renal cell carcinoma (RCC) is the most common malignant tumour in the kidney, accounting for nearly 90% of all kidney cancers [1]. Approximately 350,000 new cases of RCC were diagnosed worldwide per year, which caused more than 15,000 deaths per year in the USA and more than 140,000 deaths per year worldwide [2, 3]. RCC can have several histologic subtypes; clear cell renal cell carcinoma (ccRCC) is the most common RCC subtype in adults and accounts for approximately 70% of all RCC cases [4]. The prognosis of ccRCC varies widely. For patients with early and localized disease, the cure rate is high with a 5-year survival of more than 90%. However, 5-year survival was only 12% for patients with distant metastatic disease [5], which caused most deaths in ccRCC patients. In addition to the most basic surgical resection, many emerging therapies for the treatment of metastatic ccRCC have been proposed with improved knowledge of disease biology. Cabozantinib, an antiangiogenic agent that targets the VEGF pathway, was approved as a first-line therapy for patients with advanced ccRCC [6]. Immunotherapy, such as programmed cell death 1 (PD-1) and cell death-ligand 1 (PD-L1) blockers, has also been developed in ccRCC [7, 8]. However, each of these treatments still has some limitations. Hence, it is urgent to improve the survival of patients with ccRCC. Whereas TNM (Tumor, Node, Metastasis) staging system has been the most commonly prognostic predictive system for ccRCC patients, it does not effectively predict the aggressiveness of the ccRCC [9]. Although there are several common prognosis factors such as tumor stage, grade, and size, these factors also do not provide accurate predictions due to their molecular and genetic heterogeneity were ignored. Identifying potential valuable molecular biomarkers would enhance the prognostic value of the developed tools.

Redox homeostasis system regulates many biological processes, including cell signaling, proliferation, and differentiation by modulating intracellular antioxidant and redox signaling (ARS). Imbalances among oxidation and antioxidation can lead to oxidative stress and damage to cell functions, contributing to a variety of diseases [10, 11]. During the last decades, extensive research has revealed that disruption of the reduction–oxidation signaling can mediate cancer initiation and development by leading to molecular damage [12, 13]. Recent studies showed the imbalance of the redox homeostasis system is closely related to the RCC occurrence and progression [14, 15]. Hence, it is vital to discover potential valuable redox-related biomarkers to improve the prognostic prediction of patients with ccRCC.

In recent years, scientists have focused on molecular biomarkers in the development of a reliable prognostic biomarker in cancer [16]. The long noncoding RNA (lncRNA) is a type of noncoding RNA with transcripts of >200 nucleotides in length without any protein-coding capacity [17], but it plays important roles in the regulation of mRNA transcription and protein translation [18]. lncRNA modulated many important biological functions, such as cell growth and survival, genomic imprinting, chromatin modifications, and allosteric regulation of enzyme activities [19]. In tumor patients, abnormal expressions of lncRNA are frequent biological phenomena and closely associated with prognosis [20]. lncRNAs have been repeatedly suggested as well-accessible blood-based biomarkers in numerous urogenital malignancies, including RCC [21–23]. Therefore, the redox-related lncRNA may be used as a potential valuable biomarker or a potential therapeutic target.

In our study, we evaluated the interaction between redox and lncRNA. A nine-redox-related lncRNA signature with potential molecular prognostic value in ccRCC was identified by using both the LASSO and Cox regression analyses. We also constructed a nomogram based on this nine-redox-related lncRNA signature for improving the prognostic prediction of ccRCC patients, and it will serve as a reliable prognostic predictor tool for ccRCC patients in the future.

## 2. Materials and Methods

### 2.1. Data Sources

We searched TCGA-GDC (https://portal.gdc.cancer.gov/) for the transcriptome profiling and clinical data. We filtered the transcriptome profiling data using the following: the primary site is the kidney, the program name is TCGA, the project is TCGA-KIRC, the disease type is adenomas and adenocarcinomas, and the data category is transcriptome profiling while workflow type is HTSeq-FPKM. On the other hand, the filter criteria for clinical data included data category and format as clinical and bcr xml, respectively. We then downloaded the cart and metadata files for the transcriptome profiling data (611 samples) and the cart files for the clinical data (537 samples). The data files were decompressed and sorted into a matrix based on PERL programming. We searched the Ensembl database (http://asia.ensembl.org/index.html) for the human gene transfer format (gtf) file to transfer the gene id and annotate genes for mRNA or lncRNA. In addition, we searched GSEA-MSigDB (https://www.gsea-msigdb.org/gsea/msigdb) for the redox-related gene set by searching “redox” as keywords, and we download two redox-related gene sets as “GO_CELL_REDOX_HOMEOSTASIS” and “GO_RESPONSE_TO_REDOX_STATE.”

### 2.2. Differentially Expressed Redox-Related lncRNAs (DERRlncRNAs)

Having annotated the genes for mRNA or lncRNA, we extracted the expression of lncRNA and then used the “limma” package for the entire lncRNA data to identify the differentially expressed lncRNAs (DElncRNAs) with ∣logFC | >1 and FDR < 0.05 between tumor and normal samples. Meanwhile, we extracted the expression of redox-related gene sets then identified redox-related lncRNAs by using the Pearson Correlation Test with ∣Cor | >0.5 and p.adj < 0.001 between lncRNAs and expression of redox-related gene sets in tumor tissue. Finally, we took an intersection of DElncRNAs and redox-related lncRNAs to screen differentially expressed redox-related lncRNAs (DERRlncRNAs).

### 2.3. Random Grouping and Signature Construction

We merge the expression of DERRlncRNAs with their clinical survival data; then, all the samples were randomly split into the training and test groups at a 1 : 1 ratio. Following this, we performed univariate Cox regression of DERRlncRNAs in the training group to identify prognosis-related DERRlncRNAs with the filter criterion set at a significance of *p* < 0.05. Also, to avoid overfitting, we applied LASSO regression to screen appropriate variables from the prognosis-related DERRlncRNAs. Finally, a survival-predicting model was constructed by a multivariate Cox proportional hazard model. Importantly, a risk score formula was created based on the signature: Risk score = ∑_*i*=1_^*N*^(Exp(*i*)∙coe(*i*)). *N* is the number of redox-related lncRNA in the multivariate COX regression, Exp(i) is the expression value of lncRNA, and Coe(i) is the estimated regression coefficient of lncRNA in the multivariate Cox regression analysis. Then, the samples in both the training group and test group obtained a risk score calculated by the formula, and we set the medium value of the risk score in the training group as filter criteria that the higher risk score is high risk and the lower risk score is low risk.

### 2.4. Validation of the Survival-Predicting Model

According to the risk level judged by the risk score, we performed the Kaplan-Meier method survival analysis to test the survival-predicting availability of the signature and plot the survival curve for the samples in the training group, test group, and all group. Then, we merged the clinical data which contained age, gender, stage, and grade with the risk score of patients and rechecked to delete samples lacking accurate clinical data. Following this, we plotted the multivariate ROC curves to verify and compare the efficacy of the developed signature with the other clinical prognostic factors; the area under the curve (AUC) for multiple factors which contained age, gender, stage, grade, and risk scores was calculated and compared with each other in the training group, test group, and all samples.

### 2.5. Construction and Validation of the Risk Score-Based Nomogram

To provide clinicians with a quantitative rather than qualitative approach for predicting survival, we assembled a nomogram according to the risk score and clinicopathologic characteristics from the samples in the training group, then performed internal cross-validation, and input test group and all samples as two external validation set to perform an external validation. All the calibration curve for 1 year, 3 years, and 5 years were plotted.

### 2.6. Gene Set Enrichment Analysis (GSEA) and Clinical Correlation

Though we had tested the survival-predicting availability of the signature, how the redox-related functions worked was still unknown; thus, we divided the transcriptome file for all samples into the high-risk group and low-risk group according to the medium value of risk score in the training group and then exported the data as “cls” and “gct” format files, which were then imported into GSEA (version 4.0.3), and conducted the analysis to explore whether the redox-related functions were significantly differentially enriched between the two groups. In addition, the correlation between risk level and clinicopathologic characteristics were tested, and the differential expression of the nine redox-related lncRNAs between the high-risk and low-risk group was analyzed.

### 2.7. Coexpression Network, Correlation Plot, and Differential Expression Status

Having validated the efficacy of the nine redox-related lncRNA survival-predicting signature, we extracted the coexpression status of the redox genes and redox-related lncRNA from the primary PEARSON Correlation Test then used Cytoscape (version 3.8.0) to visualize the coexpression network. Also, the Sankey plot and correlation circle plot were used to visualize the correlation between redox genes and nine redox-related lncRNAs. In addition, the differential expression of these nine redox-related lncRNAs between normal and tumor tissue, and between different clinicopathologic characteristics were analyzed and plotted.

### 2.8. Subgroup Analysis

To further explore the correlation between risk score and clinicopathological characteristics and verify the effectiveness of the prognostic signature in different clinicopathological subgroups, all samples were divided into subgroups according to age (>65 or ≤65), gender (male or female), stage (stage I-II or stage III-IV), and grade (G1-2 or G3-4). Then, we compared the mean risk score between the different groups and performed survival analysis to validate the effectiveness of our prognostic signature in different subgroups.

### 2.9. Statistical Analysis

The data was processed using the Strawberry PERL programming language (version 5.30.2.1). All statistical analyses were performed using the R software (version 4.0.2). *p* < 0.05 was regarded as statistically significant.

## 3. Results

### 3.1. Patients and Samples

There were 611 transcriptome profiles that contained 72 normal tissues and 539 tumor tissues from 530 KIRC patients, and we took the average of the tumor samples sequenced multiple times. Also, all samples were randomly split into the training and test group at a 1 : 1 ratio, and the characteristic of the samples in the training group, test group, and all samples are shown in Table 1. Fisher's exact test was performed to compare the differences between groups. It seemed that there was no significant difference between these groups.

### 3.2. Differentially Expressed Redox-Related lncRNAs (DERRlncRNAs)

**Figure 1:**
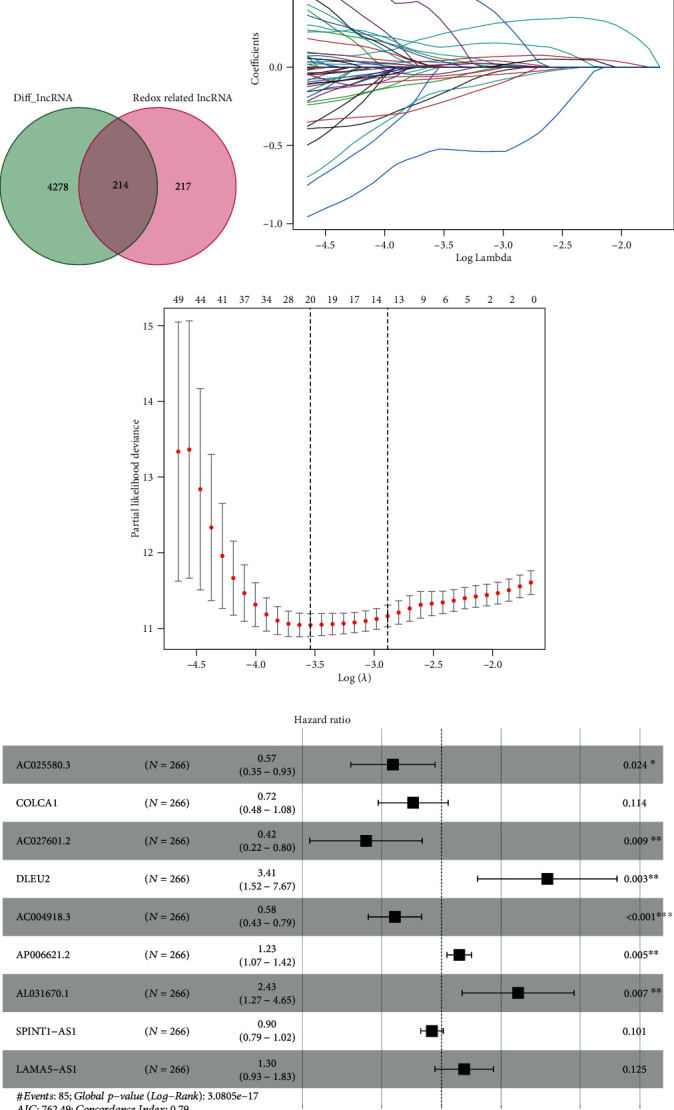
Development of the nine redox-related lncRNAs signature. (a) Screening of differentially expressed redox-related lncRNAs. (b) Variables going to zero as we increase the penalty (lambda) in the objective function of the LASSO. (c) 10-fold cross-validation for tuning parameter selection in the LASSO model, −4 < lambda.min < −3.5, and there were 20 variables (lncRNAs) left. (d) Results of the multivariate Cox proportional hazard model based on the 20 variables; nine lncRNA genes were screened to construct the signature.

As shown in Figure 1(a), there are a total of 4492 differentially expressed lncRNAs with∣logFC | >1andFDR < 0.05. And a total of 431 redox-related lncRNAs were screened with Pearson correlation coefficient ∣cor | >0.5 and p.adjust < 0.001. Then, we took an intersection of them and acquired 214 differentially expressed redox-related lncRNAs (DERRlncRNAs).

### 3.3. Construction of the Redox-Related lncRNA Survival-Predicting Signature

In the training group, we performed univariate Cox regression and got 88 significant prognostic DERRlncRNAs. Then, the LASSO regression was used to avoid overfitting and screened 20 appropriate DERRlncRNAs as variates to do the following multivariate cox regression (Figures 1(b) and 1(c)). Finally, we performed multivariate Cox regression and developed a nine-redox-related lncRNA signature containing *AC025580.3*, *COLCA1*, *AC027601.2*, *DLEU2*, *AC004918.3*, *AP006621.2*, *AL031670.1*, *SPINT1-AS1*, and *LAMA5-AS1* to predict the survival of KIRC patients (Figure 1(d)), and their detailed information is shown in Table 2. The risk score for each sample was then calculated based on the expression levels of these nine redox-related lncRNAs. Risk score = 1.23∙*DLEU*2 + 0.21∙*AP*006621.2 + 0.89∙*AL*031670.1 + 0.26∙*LAMA*5 − *AS*1 − 0.56∙*AC*025580.3 − 0.33∙*COLCA*1 − 0.87∙*AC*027601.2 − 0.54∙*AC*004918.3 − 0.11∙*SPINT*1 − *AS*1.

### 3.4. Validation of the Survival-Predicting Signature

**Figure 2:**
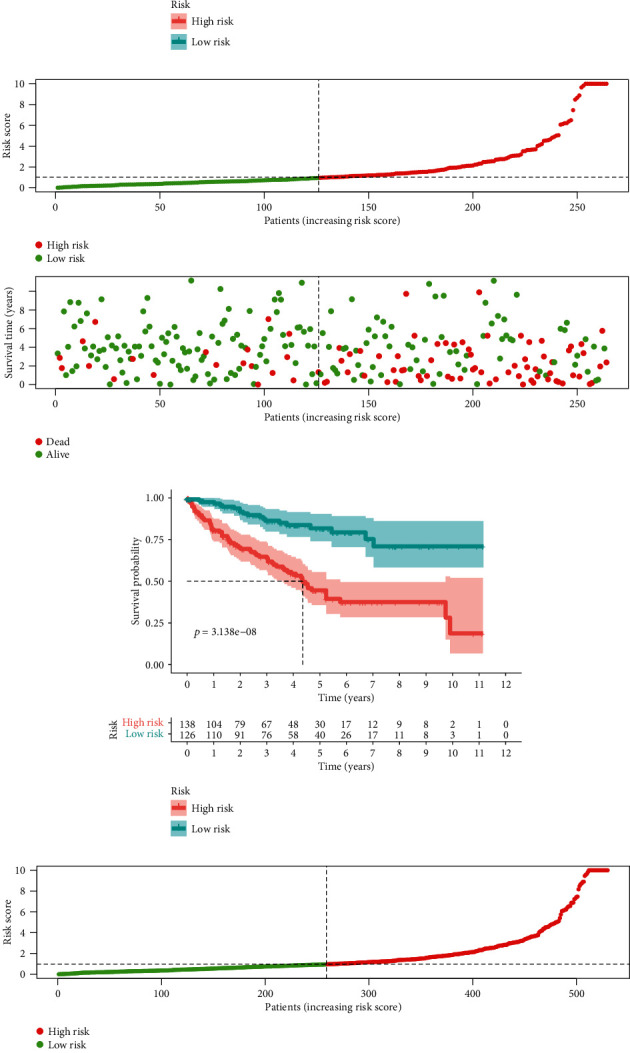
Risk plot and survival curves. (a) Risk plot of the training group. (b) Survival curve of the training group. (c) Risk plot of the test group. (d) Survival curve of the test group. (e) Risk plot of all samples. (f) Survival curve of all samples.

**Figure 3:**
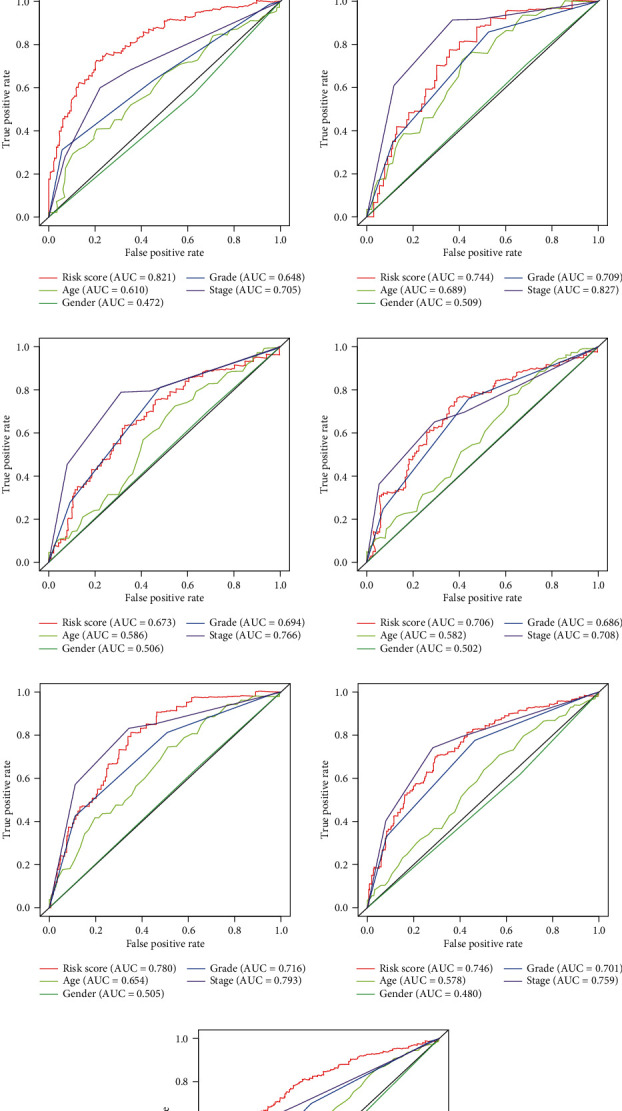
Time-dependent multivariate ROC curve. (a) One-year multivariate ROC curve in the training group. (b) Three-year multivariate ROC curve in the training group. (c) Five-year multivariate ROC curve in the training group. (d) One-year multivariate ROC curve in the test group. (e) Three-year multivariate ROC curve in the test group. (f) Five-year multivariate ROC curve in the test group. (g) One-year multivariate ROC curve in all samples. (h) Three-year multivariate ROC curve in all samples. (i) Five-year multivariate ROC curve in all samples.

Having developed the nine redox-related lncRNA signature, all the samples both in the training group and test group acquired a risk score, and we set the medium value of the risk score in the training group as the cutoff to judge the risk level of patients as high risk or low risk (Figures 2(a), 2(c), 2(e)). Following this, survival analysis was performed to verify the survival-predicting availability of the signature (Figures 2(b), 2(d), 2(f)). Time-dependent ROC curve for 1 year, 3 years, and 5 years in the training group (Figures 3(a)–3(c)), test group (Figures 3(d)–3(f)), and all samples (Figures 3(g)–3(i)) were drawn, and the AUC for the risk score in these three groups showed that risk score could act as an efficient prognostic factor even compared with other commonly used clinical prognostic factor.

### 3.5. Construction and Validation of the Risk Score-Based Nomogram

**Figure 4:**
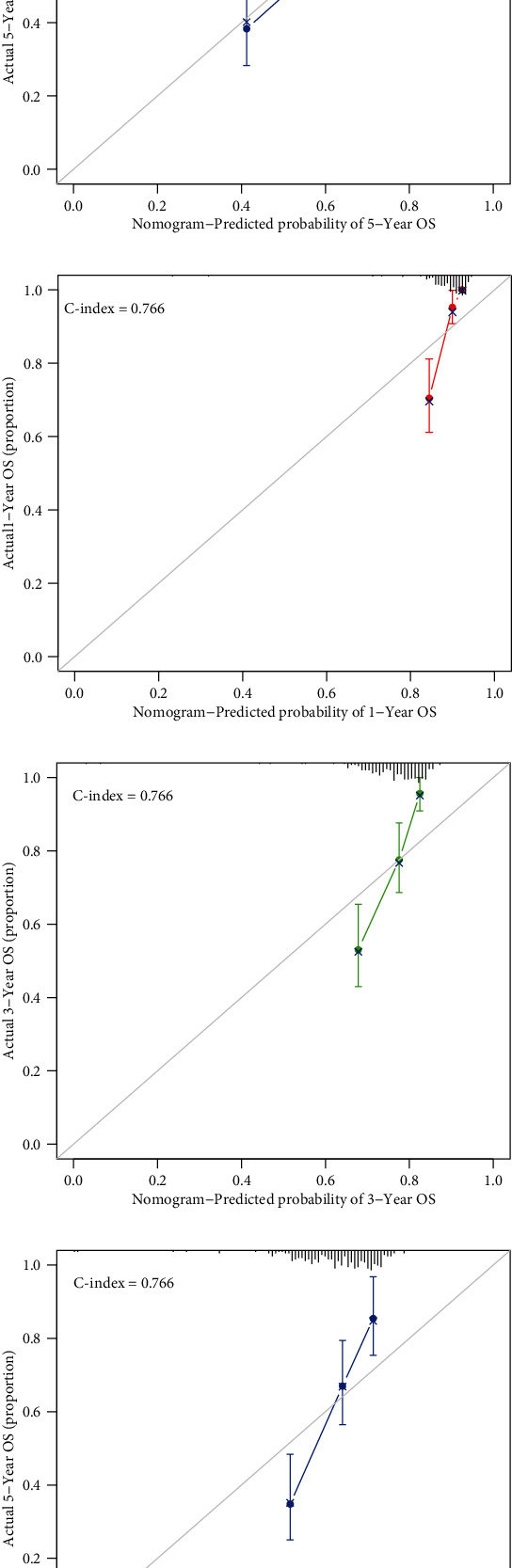
Nomogram and calibration curves. (a) Prognostic nomogram assembled from the training group to predict survival for ccRCC patients. (b, c, d) Calibration curves for the nomogram at 1-year, 3-year, and 5-year periods in the training group. (e, f, g) Calibration curves for the nomogram at 1-year, 3-year, and 5-year periods in the test group. (h, i, j) Calibration curves for the nomogram at 1-year, 3-year, and 5-year periods in all samples.

Having verified the efficacy of the signature, we would like to develop a more quantitative rather than qualitative approach for clinicians to predict the survival of the KIRC patients. Thus, we assembled a nomogram according to the risk score and clinicopathologic characteristics that contained age, gender, stage, and grade from the samples in the training group to predict the survival rate for 1 year, 3 years, and 5 years (Figure 4(a)). Also, the calibration curve for 1-year, 3-year, and 5-year survival rate in the training group (Figure 4(b)–4(d)), test group (Figure 4(e)–4(g)), and all KIRC samples (Figure 4(h)–4(j)) were plotted. Also, C-index was calculated to assess the performance of the nomogram assembled according to the training group, and that was 0.782, 0.766, and 0.774 in the training group, test group, and all samples, which showed the perfect performance of the nomogram.

### 3.6. GSEA and Clinical Correlation

**Figure 5:**
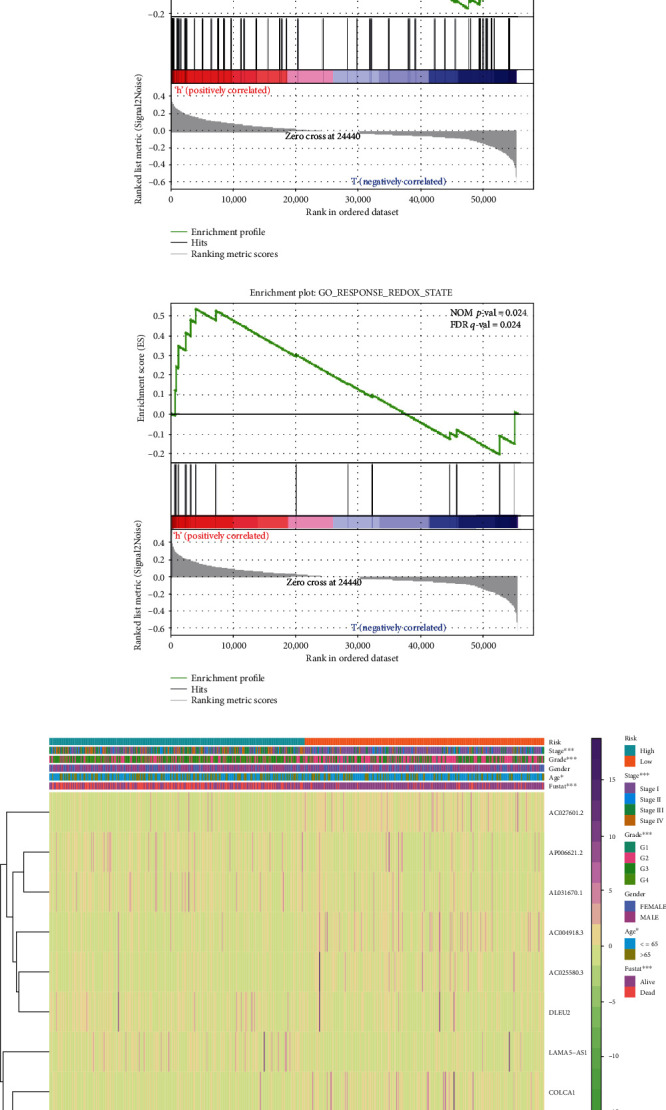
Gene set enrichment analysis (GSEA) of high-risk and low-risk ccRCC patients based on the redox-related lncRNA prognostic signature and expression heat map between high/low-risk patients. (a, b) The GSEA results show a significant enhancement of redox-related functions in the high-risk ccRCC patients. (c) Expression heat map for the nine redox-related lncRNAs in all samples.

To explore the different redox-related functions in the high/low-risk group, we performed the enrichment analysis by using GSEA version 4.0.3 as shown in Figures 5(a) and 5(b). It showed that both two redox-related functions were enhanced in the high-risk group, of which the GO term response to the redox state was significantly enhanced in the high-risk group (NOM *p* value = 0.024, FDR *q*-value = 0.024), while the other GO term cell redox homeostasis was not significant (NOM *p* value = 0.065, FDR *q* value = 0.065). In addition, the correlation between risk level and clinical characteristics and the differential expression of the nine redox-related lncRNAs in high/low risk were analyzed as shown Figure 5(c). It seemed age, grade, and stage were all significantly related to the risk level, which was also consistent with the outcome that high risk resulted in high mortality.

### 3.7. Coexpression Network, Correlation Plot, and Differential Expression Status

**Figure 6:**
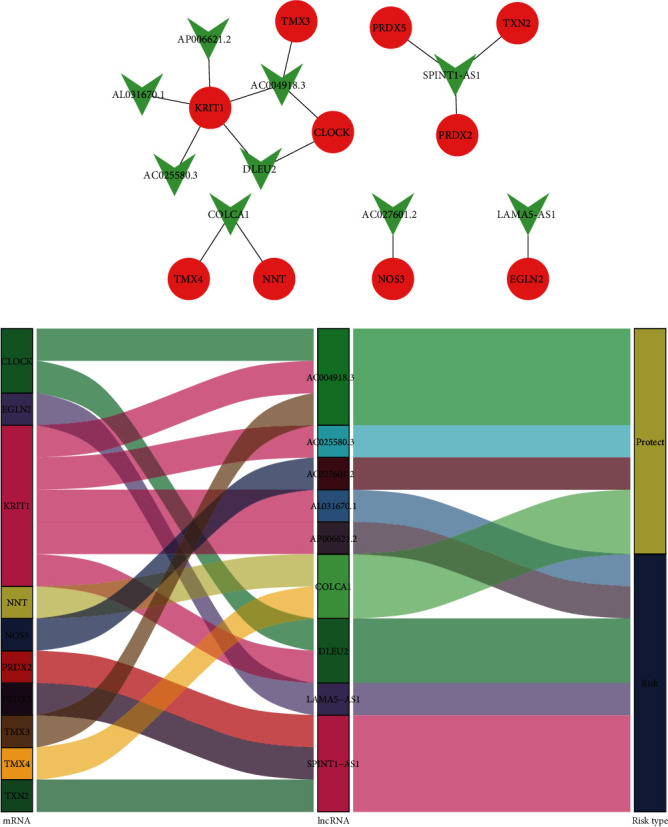
Redox gene-redox-related lncRNA interaction network and Sankey plot. (a) The interaction network between the redox-related lncRNA genes and redox-related protein-coding genes: red circle for redox-related mRNAs and green V-shape for redox-related lncRNAs. (b) In the Sankey plot for the interaction network, four of these redox-related lncRNAs were protective while the other five were indicative of risk.

**Figure 7:**
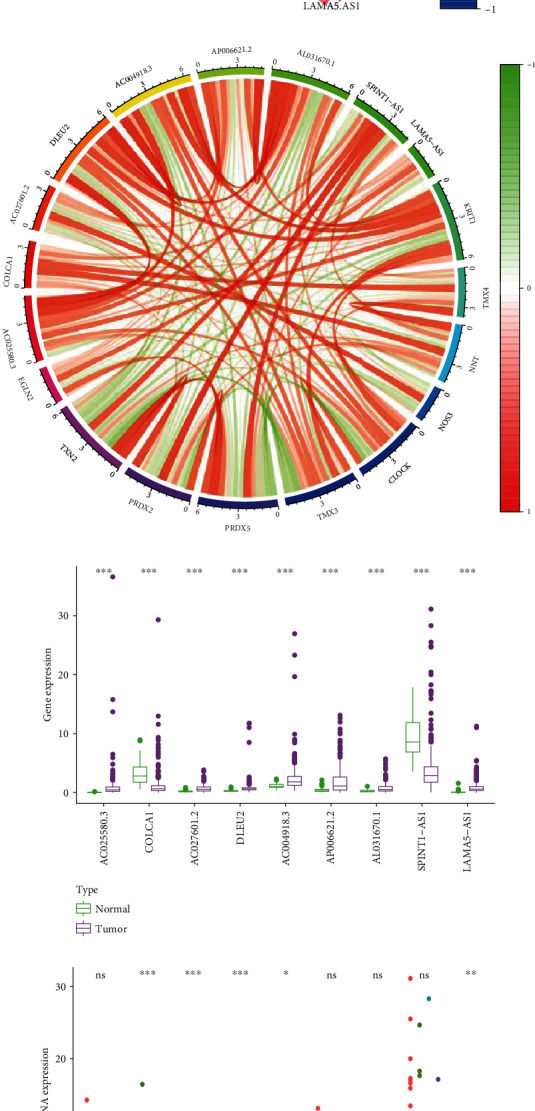
Correlation plot and the differential expression between normal/tumor tissue. (a) Correlation network for the redox gene and redox-related lncRNAs. (b) Circle plot for the correlation between redox gene and redox-related lncRNAs. (c) Differential expression for the nine redox-related lncRNAs between normal/tumor tissue. (d) Differential expression for the nine redox-related lncRNAs between stage I/II/III/IV patients.

Finally, we focused on these nine redox-related lncRNAs about their coexpression and differential expression. The redox gene-redox-related lncRNA coexpression network was constructed (Figure 6(a)), and the Sankey plot (Figure 6(b)) showed that 4 of them were protective and the other 5 were risky. In addition, the correlation between the redox gene and redox-related lncRNA was plotted as the correlation network (Figure 7(a)) and correlation circle plot (Figure 7(b)). The differential expression status of the nine redox-related lncRNAs between normal/tumor tissue as in Figure 7(c) showed that 7 of 9 redox-related lncRNAs were significantly high expression in the tumor tissue while the 2 left were significantly low expression in the tumor tissue. Besides, the correlation between the expression of these nine redox-related lncRNAs and the clinicopathological staging was explored and shown in Figure 7(d). As for the differential expression status of the nine redox-related lncRNAs in different age, gender, and grade, the boxplot is shown in the supplementary file (available here).

### 3.8. Subgroup Analysis

**Figure 8:**
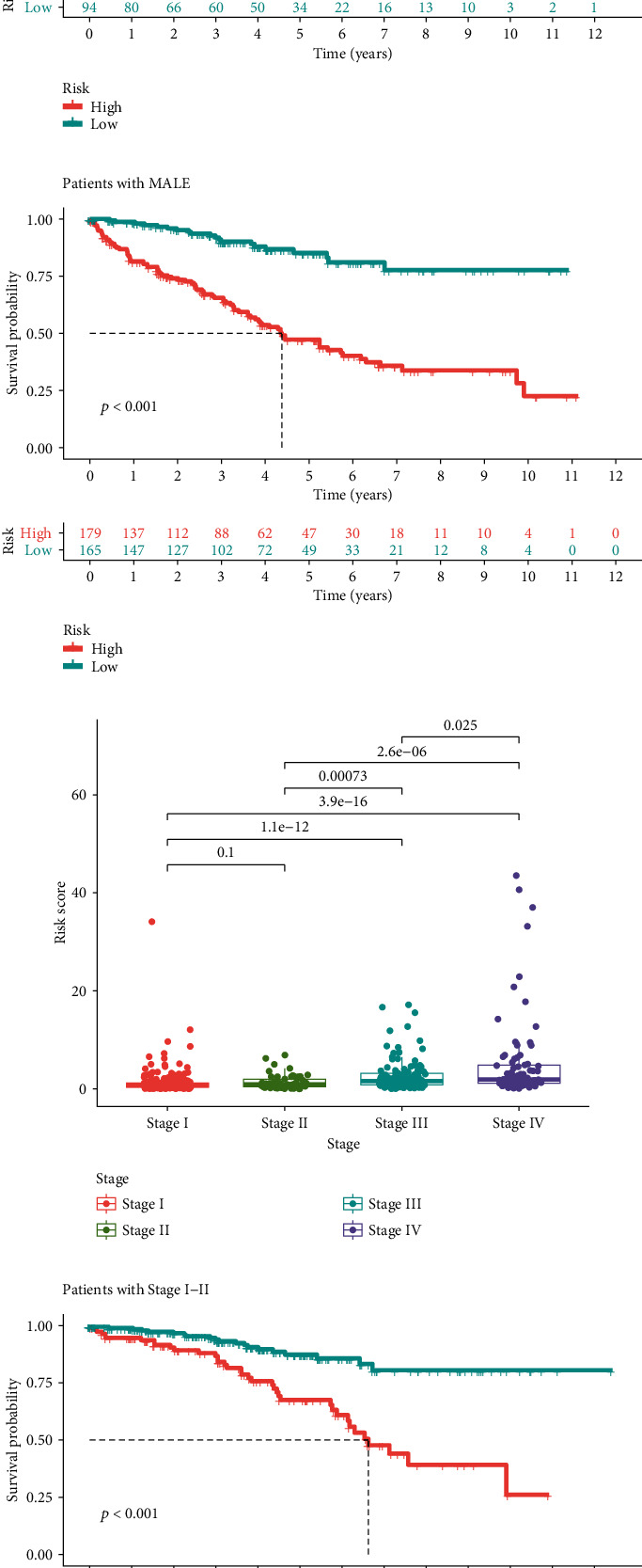
Difference in risk score between subgroups and further subgroup survival analysis. (a) Difference of risk score in patients with age ≤ 65 or >65. (b) Risk score-based survival analysis in patients with age ≤ 65. (c) Risk score-based survival analysis in patients with age > 65. (d) Difference of risk score in female or male patients. (e) Risk score-based survival analysis in female patients. (f) Risk score-based survival analysis in male patients. (g) Difference of risk score in patients with different stage. (h) Risk score-based survival analysis in patients with stage I-II. (i) Risk score-based survival analysis in patients with stage III-IV. (j) Difference of risk score in patients with different grade. (k) Risk score-based survival analysis in patients with G1-2. (l) Risk score-based survival analysis in patients with G3-4.

In age subgroups, risk score in patients with age > 65 was significantly higher than patients with age ≤ 65 (Figure 8(a)), and the prognostic signature was verified effective in both age ≤ 5 (Figure 8(b)) and age > 65 (Figure 8(c)) subgroups. In the gender subgroup, there was no significant difference in the risk score between female and male patients (Figure 8(d)). And the prognostic signature was also effective in both female (Figure 8(e)) and male (Figure 8(f)) subgroups. As for the stage subgroups (Figure 8(g)), the risk score in stage III was significantly higher than that in stage II, and stage IV was significantly higher than stage III. However, stage II was higher than stage I with no significant difference. And the prognostic signature was effective in both the stage I-II (Figure 8(h)) and stage III-IV (Figure 8(i)) subgroups. In the grade subgroups, the risk score was significantly increased between G1 and G2 and G3 and G4 (Figure 8(j)), and the prognostic signature was still effective in both the G1-2 (Figure 8(k)) and G3-4 (Figure 8(l)) subgroups.

## 4. Discussion

ccRCC is the most common type of RCC in humans. With the development of clinical management of ccRCC, several prognostic factors, such as tumor grade and stage, tumor size, and tumor number, are well characterized. However, ccRCC has complex genetic and molecular alterations [24], which could affect the biological processes, and some of the biological processes are closely associated with the prognosis of ccRCC patients, such as autophagy [25], ferroptosis [15], and redox [26]. Most of these commonly used prognostic factors do not consider either genetic and molecular alterations or dysregulated biological processes, and it made these commonly used prognostic factors not perfect for accurate prognostic prediction of ccRCC patients [27]. As an emerging genetic and molecular biomarker, lncRNA is a new class of noncoding RNA molecules that regulate cancer cell growth, progression, and survival [28]. Therefore, it is necessary to establish a lncRNA signature to predict the prognosis of ccRCC patients.

In this study, we focused on the redox process and constructed a nine redox-related lncRNA prognostic signature (Risk score = 1.23∙*DLEU*2 + 0.21∙*AP*006621.2 + 0.89∙*AL*031670.1 + 0.26∙*LAMA*5 − *A*S1 − 0.56∙*AC*025580.3 − 0.33∙*COLCA*1 − 0.87∙*AC*027601.2 − 0.54∙*AC*004918.3 − 0.11∙*SPINT*1 − *AS*1.) in the training group by the LASSO regression and COX regression, which considered both molecular alteration and dysregulated biological process. Meanwhile, *χ*^2^-test or Fisher's exact test found the nine redox-related lncRNA signature was significantly related to tumor grade, stage, patients' age, and survival status of ccRCC patients. In addition, risk analysis, survival analysis, and 1-year, 3-year, 5-year multivariate ROC in both the training group and test group well verified the efficacy of the survival-predicting signature. Then, a concise nomogram consisted of the nine redox-related lncRNA signature, age, gender, grade, and stage was developed from the data in the training group for prognostic prediction of ccRCC patients; both internal cross-validation and external set validation showed great effectiveness, and the calibration curve showed great convergency to the standard curve. Further subgroup analysis verified the effectiveness of our prognostic signature and indicated the universality of this prognostic signature. Finally, having verified the effectiveness of our nine-redox-related lncRNA signature, we focused on the interaction between the redox genes and these nine redox-related lncRNAs, constructed a redox gene-lncRNA interaction network, and performed a GSEA analysis to explore the differences in redox functions between high/low risk. Interestingly, we found both GO CELL REDOX HOMEOSTASIS and GO RESPONSE TO REDOX STATE were enhanced in the high-risk group, which was consistent with the previous study that high redox level in cancer could influence the survival of tumor patients by initiating/stimulating tumorigenesis and supporting transformation/proliferation of cancer cells or causing cell death [29].

In this signature which consisted of nine prognostic lncRNAs related to redox genes, *COLCA1* has been reported and identified as a key lncRNA in colorectal cancer [30–32], and *DLEU2* has been reported related to the development of multiple cancers [33–36]. Chen et al. reported that lncRNA *DLEU2* could regulate *miR-30a-5p* and related to the aggressiveness of ccRCC [37]. *SPINT1-AS1* has been reported as a prognostic factor in esophageal squamous cell carcinoma and colorectal cancer [38, 39]. Xiang et al. also reported *SPINT1-AS1* as a crucial factor for pan-cancer cell sensitivity to lapatinib [40]. *LAMA5-AS1* has been reported as a significant factor in the pathogenesis of multiple myeloma [41]. As for the other 5 lncRNAs, there were few reports about them.

Redox plays an essential role in the pathogeneses and progression of tumors. Regulation of reactive oxygen species (ROS) production is crucial in highly proliferative cancer cells, owing to the presence of oncogenic mutations that promote aberrant metabolism and gene expression [42]. Cancer cells can produce ROS, which diffuses into the tumor microenvironment, then initiates stromal oxidative stress and autophagy, and leads to angiogenesis. Sosa et al. [43] revealed that cancer cells develop resistance to ROS by inducing a new redox balance, which further results in cellular adaptation and proliferation under increased oxidative pressure. Indeed, the imbalance of the redox homeostasis system is closely related to the RCC occurrence and progression. In RCC patients, cytosolic antioxidant enzyme activities are shown to be decreased [44]. In recent years, redox balance has been reported regulated by long noncoding RNAs [45]; more and more researchers developed to identify the significant lncRNA-redox regulation network. Chen et al. reported that lncRNA *GAS5* regulated the redox balance and dysregulates the cell cycle and apoptosis in malignant melanoma cells [46]. He et al. reported lncRNA *MACC1-AS1* promoted stemness and chemoresistance through fatty acid oxidation in gastric cancer [47]. Here, our works contributed to further the comprehension of these nine redox-related lncRNAs and their interaction with redox balance, which might provide potential targets for the treatment in the future.

However, our study still has some limitations. First, the training group and test group are both obtained from TCGA, and it would be better if there is an independent cohort as an external validation set. In addition, we did not define the mechanisms behind the lncRNA-based signature's mediation of redox in the initiation and progression of ccRCC. Despite these limitations, this is the first redox-related lncRNA-based survival-predicting signature, and our nomogram provides a quantitative approach for clinicians to predict survival, which can easily separate patients with poor prognosis from all the ccRCC patients by performing PCR. Then, clinicians can perform more individualized treatment regimens for patients with different prognosis, which will contribute to individual treatment and save more public health resources. Meanwhile, this nomogram consisted of objective indicators, which can reduce the interobservers' differences and more accurately predict survival.

## 5. Conclusions

In summary, we successfully developed and verified a nine-redox-related lncRNA signature that could predict the overall survival of ccRCC patients. The prognostic signature proved superior compared to the other common prognostic factors. We further assembled a nomogram connecting this signature with clinicopathologic characteristics for 1-, 3-, and 5-year OS, which can provide clinicians with a quantitative rather than qualitative approach in predicting ccRCC survival. This will help clinicians make treatment decisions more easily and accurately in the future. It is, however, necessary to carry out a large-scale, multicenter prospective research to confirm our results.

## Figures and Tables

**Table 1:** Clinical characteristics of the KIRC patients.

	Overall	Test	Train	*p*
*n*	530	264	266	
Age (mean (SD))	60.56 (12.14)	60.48 (11.76)	60.65 (12.52)	0.875
Gender = female/male (%)	186/344 (35.1/64.9)	82/182 (31.1/68.9)	104/162 (39.1/60.9)	0.065
Grade (%)				0.432
G1	14 (2.6)	5 (1.9)	9 (3.4)	
G2	227 (42.8)	108 (40.9)	119 (44.7)	
G3	206 (38.9)	110 (41.7)	96 (36.1)	
G4	75 (14.2)	36 (13.6)	39 (14.7)	
GX	5 (0.9)	4 (1.5)	1 (0.4)	
Unknown	3 (0.6)	1 (0.4)	2 (0.8)	
Stage (%)				0.169
Stage I	265 (50.0)	125 (47.3)	140 (52.6)	
Stage II	57 (10.8)	26 (9.8)	31 (11.7)	
Stage III	123 (23.2)	68 (25.8)	55 (20.7)	
Stage IV	82 (15.5)	45 (17.0)	37 (13.9)	
Unknown	3 (0.6)	0 (0.0)	3 (1.1)	
T (%)				0.414
T1	21 (4.0)	10 (3.8)	11 (4.1)	
T1a	140 (26.4)	68 (25.8)	72 (27.1)	
T1b	110 (20.8)	49 (18.6)	61 (22.9)	
T2	55 (10.4)	27 (10.2)	28 (10.5)	
T2a	10 (1.9)	3 (1.1)	7 (2.6)	
T2b	4 (0.8)	1 (0.4)	3 (1.1)	
T3	5 (0.9)	2 (0.8)	3 (1.1)	
T3a	120 (22.6)	69 (26.1)	51 (19.2)	
T3b	52 (9.8)	30 (11.4)	22 (8.3)	
T3c	2 (0.4)	0 (0.0)	2 (0.8)	
T4	11 (2.1)	5 (1.9)	6 (2.3)	
M (%)				0.283
M0	420 (79.2)	209 (79.2)	211 (79.3)	
M1	78 (14.7)	43 (16.3)	35 (13.2)	
MX	30 (5.7)	12 (4.5)	18 (6.8)	
Unknown	2 (0.4)	0 (0.0)	2 (0.8)	
N (%)				0.68
N0	239 (45.1)	124 (47.0)	115 (43.2)	
N1	16 (3.0)	8 (3.0)	8 (3.0)	
NX	275 (51.9)	132 (50.0)	143 (53.8)	
Risk = high/low (%)	271/259 (51.1/48.9)	138/126 (52.3/47.7)	133/133 (50.0/50.0)	0.663

**Table 2:** The detailed information of the nine redox-related lncRNAs used to construct the prognostic signature.

Gene symbol	Ensemble ID	Gene_biotype	Coef
AC025580.3	ENSG00000275672	Antisense (lncRNA)	-0.56136127
COLCA1	ENSG00000196167	Antisense (lncRNA)	-0.326969031
AC027601.2	ENSG00000262115	Antisense (lncRNA)	-0.873174537
DLEU2	ENSG00000231607	Antisense (lncRNA)	1.228067267
AC004918.3	ENSG00000270157	Sense intronic	-0.539291321
AP006621.2	ENSG00000255142	lincRNA	0.207380745
AL031670.1	ENSG00000275582	Antisense (lncRNA)	0.889174399
SPINT1-AS1	ENSG00000261183	Antisense (lncRNA)	-0.10982088
LAMA5-AS1	ENSG00000228812	Antisense (lncRNA)	0.264867342

Notes: Antisense: transcripts that overlap the genomic span (i.e., exon or introns) of a protein-coding locus on the opposite strand. Sense intronic: a long noncoding transcript in introns of a coding gene that does not overlap any exons. lincRNA (long intergenic ncRNA): transcripts that are long intergenic noncoding RNA locus with a length > 200 bp. Requires lack of coding potential and may not be conserved between species.

## Data Availability

The source data of this study were derived from the public repositories, as indicated in the section of “Materials and Methods” of the manuscript. And all data that support the findings of this study are available from the corresponding author upon reasonable request.

## Supplementary Materials

Supplementary Materials Supplementary file. Differential expression of the nine redox-related lncRNAs in different groups. S1. Differential expression between age ≤ 65 and age > 65. S2. Differential expression between male and female. S3. Differential expression between different pathological grade.
