# Supplementary material for: Development and Validation of a Nine-Redox-Related Long Noncoding RNA Signature in Renal Clear Cell Carcinoma
Source: Oxid Med Cell Longev. 2020 Dec 28;2020:6634247. doi: 10.1155/2020/6634247 (PMC7781722; doi:10.1155/2020/6634247)

Supplementary file. Differentially expression of the nine redox related lncRNAs in different groups.

S1. Differentially expression between Age ≤65 and Age >65

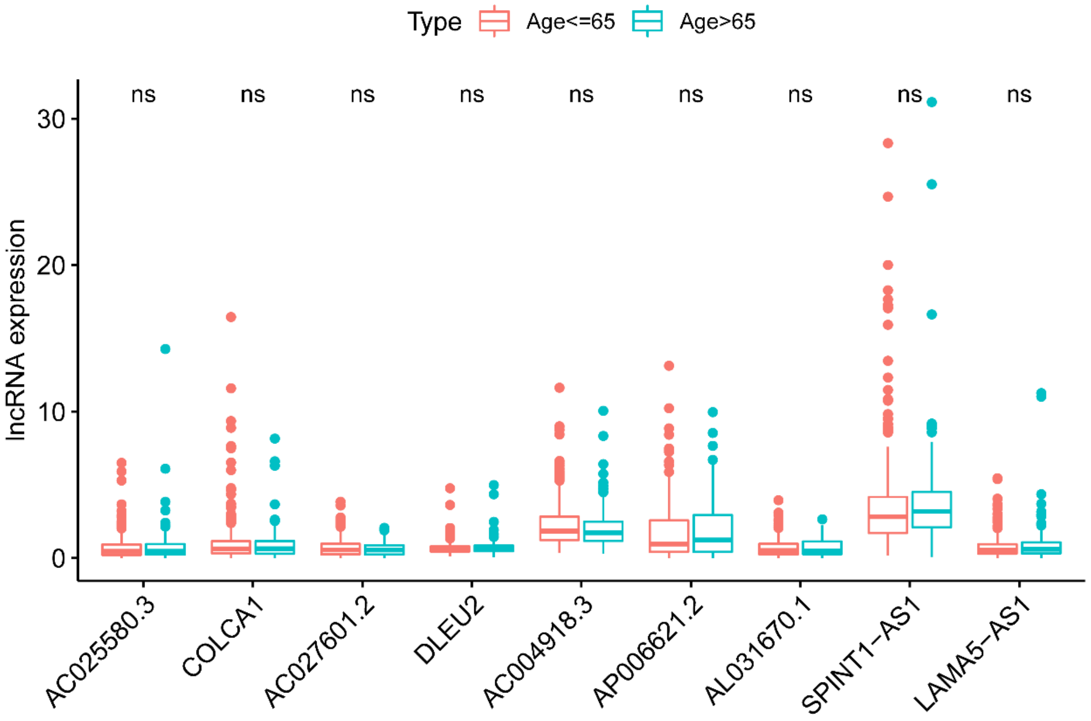

S2. Differentially expression between male and female

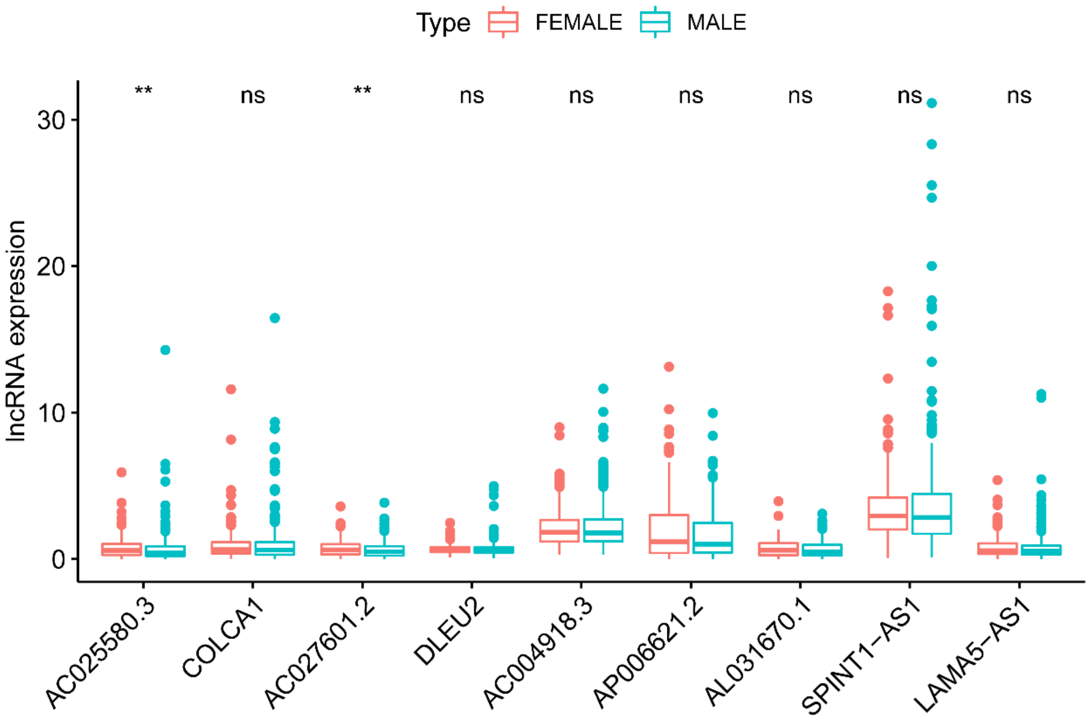

S3. Differentially expression between different pathological grade

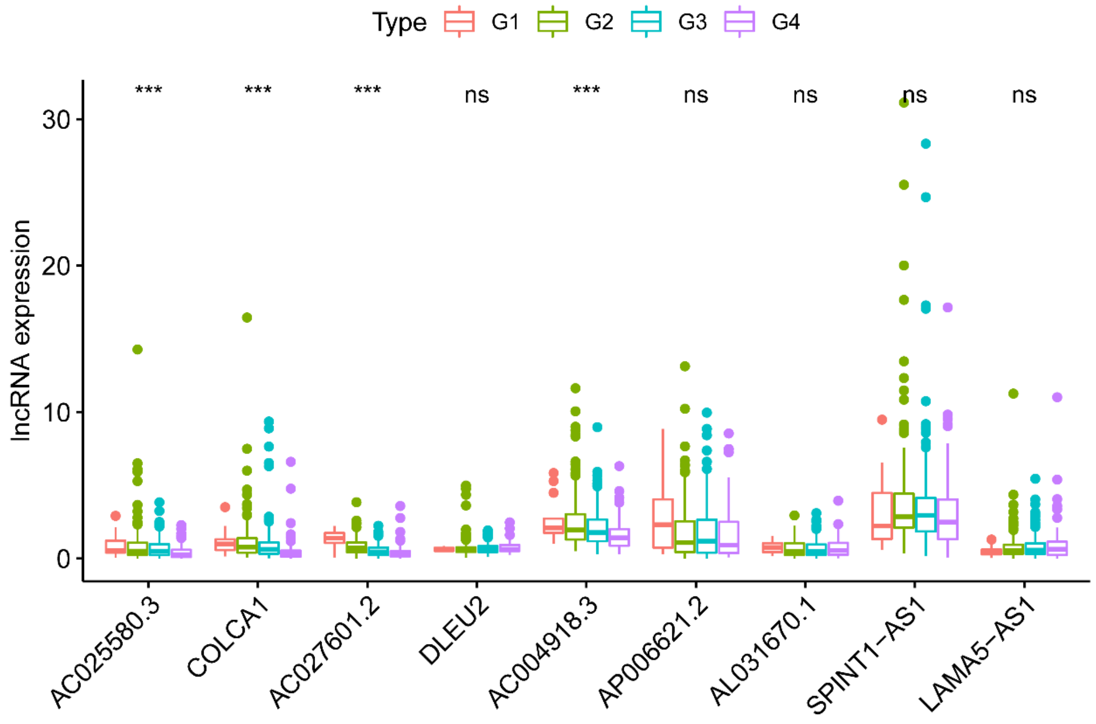

Supplement: Supplementary Materials — Supplementary file. Differential expression of the nine redox-related lncRNAs in different groups. S1. Differential expression between age ≤ 65 and age > 65. S2. Differential expression between male and female. S3. Differential expression between different pathological grade. [file 6634247.f1.pdf]
